# Mid-Wave Infrared Polarization Combiner Based on Reflective Metasurface

**DOI:** 10.3390/mi17010036

**Published:** 2025-12-28

**Authors:** Lulu Yang, Xin Wang, Xuhui Li, Liquan Dong

**Affiliations:** 1 School of Optics and Photonics, Beijing Institute of Technology, Beijing 100081, China; luluyang0402@163.com (L.Y.); lixuhui2023@126.com (X.L.); kylind@bit.edu.cn (L.D.); 2 Beijing Key Laboratory for Precision Optoelectronic Measurement Instrument and Technology, Beijing Institute of Technology, Beijing 100081, China; 3 Key Laboratory of Photonics Information Technology, Ministry of Industry and Information Technology, Beijing 100081, China

**Keywords:** polarization beam combining, mid-wave infrared, metasurface

## Abstract

Polarization beam combining (PBC) is an important technology for enhancing laser brightness. The conventional bulk polarization beam combiners are Brewster plates and birefringent polarization prisms. However, in the mid- and long-wave infrared range, the beam combining performance is limited by the transmission and birefringent coefficient of the available materials. In this paper, a polarization beam combiner based on a reflection metasurface was proposed. The phases of incident beams with orthogonal linear polarizations were individually manipulated by the side lengths of the rectangular silicon pillar. A metasurface polarization beam combiner operating band was designed and fabricated. When the two beams at 4.6 μm with orthogonal linear polarizations were incident on the metasurface at angles of −13.3° and 13.3°, respectively, they were reflected in the 0°-direction. The overall beam combining efficiency was 88.9%. When both of the quantum cascade lasers used in the experiments were in the fundamental transverse Gaussian mode, the measured beam quality factors *M^2^* of the combined beam were 1.21 and 1.14 along the fast and slow axes, respectively. Both simulation and experimental results demonstrated that the proposed metasurface was an efficient polarization beam combiner with negligible wavefront distortion. It is a promising alternative to traditional bulk optics for the mid- and long-wave infrared.

## 1. Introduction

Mid- and long-wave infrared lasers play a critical role in a wide range of fields, from spectroscopy and environmental monitoring to defence, medical applications, materials processing, and fundamental scientific research [1]. The most commonly used lasers in mid- and long-wave infrared are gas lasers, optical parametric oscillator and quantum cascade laser (QCL). Owing to the extensive design flexibility and compact structure, QCL has attracted significant attention since its invention in 1994 [2,3]. However, limited by the upper-level injection efficiency, the reported output power of a mid-infrared QCL operating on a fundamental transverse Gaussian mode was less than 5 W currently. It is a great challenge to further increase the output power by optimizing the active layer structures. As we all know, high power/energy and good beam quality can improve the performance in these application fields [4,5]. The development history of near-infrared semiconductor lasers reveals that incoherent beam combining would be a pivotal technique for boosting the output power of QCL [6,7,8]. Among the most important three technologies of spectral beam combining [9,10,11], polarization beam combining (PBC) and spatial beam combining [12], PBC takes full advantage of the linear polarization characteristic of semiconductor lasers, offering the benefits of a simple structure and high combining efficiency. Furthermore, PBC can be applied with spatial beam combining and spectral beam combining to further enhance the laser power and brightness.

Currently, commercial polarization combiners include coated Brewster plate [13] and birefringent polarization prism [14]. In 2008, Wagner et al. fabricated a silicon Brewster plate in the 3.7–5 μm spectral range [13]. The coating on the S-light reflection surface exhibited high reflectivity for S-polarization and high transmittance for P-polarization. The combining efficiency was 82%. To improve the combining efficiency critical and complicated multi-layer coating has to be applied. Additionally, the large Brewster angle introduced challenges in optical alignment. In 2025, Zhang et al. invented a Hg_2_Cl_2_ birefringent prism [14]. The working spectral range covered both mid- and long-wave infrared. The prism was used to combine the beams with orthogonal linear polarizations at 4.6 μm. The efficiency of 81% was obtained without surface coating. The predominant loss was mainly induced by the Fresnel reflection. To improve the beam combining efficiency, the polarization-dependent anti-reflection coatings had to be deposited on both the incident and output prism surfaces.

Compared with conventional polarization beam combiners, metasurface offers more flexible phase and polarization state manipulating abilities [15,16,17]. Optical absorbers [18,19,20], optical imaging [21,22], and multi-path beam splitters [23,24] have been successfully demonstrated. In 2015, Jia et al. studied a metasurface-based polarization beam splitter operating in the 8.4–9.6 GHz [25]. The beam splitting efficiency was 75%. As a reversible optical device, it can also function as a polarization beam combiner. In 2023, Chen et al. proposed a spectro-polarimetric beam splitter in the 3–5 μm band [26]. The incident light was split into five spectral channels and two polarization channels. The optical efficiency of the splitter was 80%. These works provided new insights for the polarization beam combiner design.

In our study, a polarization beam combiner based on a reflective metasurface was investigated. To obtain high reflection in mid-infrared, the unit cell of the metasurface was composed of a high-refractive-index amorphous silicon antenna, a low-refractive-index silica middle layer, and a high-reflective gold film. The high-refractive-index amorphous silicon provided not only the large phase manipulating range with the relatively low pillar height but also strong field resonance to minimize the loss. In addition, the optical field was tightly confined within and around the antennas. Therefore, the unit cell can be treated independently to synthesize the target wavefront precisely. The supercell of the metasurface comprised 10 discrete square unit cells with a side length of 2 μm. When the X-linearly polarized (XLP) and Y-linearly polarized (YLP) lasers with 4.6 μm central wavelength were incident on the metasurface with −13.3° and 13.3°, respectively, the reflection angles of both incident beams were zero degrees. Through optimizing the antennas’ geometric parameters and the layer thicknesses, high reflectance of 97.8% and 98.4% was obtained for the XLP and YLP beams, respectively. Based on the metasurface, we constructed a PBC setup. Experimental results showed that the beam quality factors (*M*^2^) of the combined beam were 1.21 and 1.14 along the fast and slow axes of the incident QCLs, respectively. The beam combining efficiency was 88.9%. The high efficiency did not depend on the complicated coatings, which made the metasurface suitable for high-power applications. In addition, the metasurface-based polarization beam combiner showed a high efficiency crossing a broad bandwidth of 500 nm. The broadband characteristics held the potential for spectro-polarimetric beam combining of multiple input beams, which could significantly enhance the power and brightness of mid-wave infrared lasers.

## 2. The Design Principle of the Metasurface Polarization Beam Combiner

**Figure 1:**
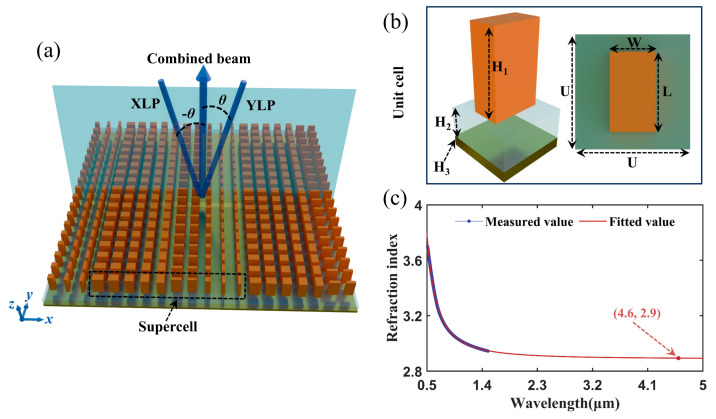
Structure of the metasurface polarization beam combiner. (**a**) Schematic of the metasurface polarization beam combiner, (**b**) the prototype of the unit cell, (**c**) the refractive index of the amorphous silicon.

Metasurfaces offer flexible control on beam propagation by phase shift manipulation on the sub-wavelength scale. Figure 1a is the schematic of the proposed reflection metasurface. When the XLP and YLP lasers were incident on the metasurface with symmetric angles, they were combined coaxially. The reflective plane of the metasurface polarization beam combiner is the *xoz* plane. The symmetric optical path configuration helped to simplify optical path alignment complexity. To achieve beam combining by the reflection of both incident beams, the spatial phase-shift distribution of the metasurface-based polarization beam combiner has to satisfy the generalized Snell’s law of reflection [15,27,28]

(1) $$\left\{\begin{array}{l} \frac{d\varphi_{\mathrm{XLP}}}{dX}=\frac{2\pi }{\lambda }\sin\theta \\ \frac{d\varphi_{\mathrm{YLP}}}{dX}=-\frac{2\pi }{\lambda }\sin\theta \end{array}\right.,$$

where *θ* and −*θ* denote the incident angles for the XLP and YLP incident lights, respectively. $\varphi_{XLP}$ and $\varphi_{YLP}$ are phase shifts induced by the metasurface structures, and *λ* is the wavelength of the incident light. According to Equation (1), if the phase gradients of the metasurface were designed to be −2*π*sin*θ*/*λ* and 2*π*sin*θ*/*λ*, respectively, for the two orthogonal polarized lights [29], both beams were reflected towards the metasurface normal. Therefore, the two incident beams were combined coaxially.

The metasurface was composed of periodically arranged supercells, which contained *N* discrete unit cells. Figure 1b shows the prototype of the unit cell with the side length of *U*. The unit cell consists of a bottom metal layer, an intermediate dielectric spacer, and a top rectangular pillar. Thus, *N·U* represented the period of the supercell along the *x*-direction. The phase shift of the supercell should cover an integer multiple of 2*π* (360°) to avoid the phase discontinuities between adjacent supercells.

To realize PBC, the phase shifts of the two orthogonally linearly polarized incident lasers were independently controlled by adjusting the length (L) and width (W) of the rectangular pillars. In this study, *N* was 10. The phase shift difference between adjacent unit cells was 36°. Assuming the central wavelength of the incident laser was 4.6 μm, the unit cell period *U* was chosen to be 2 μm to prevent the resonant coupling between adjacent unit cells. According to Equation (1), the calculated incident angles for the XLP and YLP lights were −13.3° and 13.3°, respectively.

To obtain optical resonance and low propagation loss, the materials of the bottom metal, intermediate dielectric and top rectangular antennas were chosen to be Au, silicon dioxide (SiO_2_) and silicon, respectively.

Due to the fabrication challenges associated with monocrystalline silicon, the top silicon pillars were fabricated by amorphous silicon (α-Si). However, the PECVD growth of α-Si with conventional parameters typically yields a refractive index around 2.0. To achieve the 0 to 360° phase shift coverage, the height of the pillar might be larger than 4 μm. It will induce undesired loss and electric field coupling between the neighbouring unit cells. To increase the refractive index of α-Si, a higher silicon substrate temperature (350 °C) was employed in our deposition. It could enhance the surface migration of silicon atoms, thereby promoting the formation of a denser network structure to increase the refractive index. In addition, Argon was used as the diluent gas for silane. In conjunction with low RF power and a low dilution ratio, sufficient energy for precursor decomposition was provided, and the film density was improved. After deposition, high-temperature annealing (600 °C) was conducted to facilitate structural relaxation within the film, which could reduce the dangling bonds and micro-voids, and significantly enhance the density and refractive index. By using the optimized process parameters, the refractive index of α-Si film was increased to be close to that of crystalline silicon.

The refractive index of α-Si film was measured using the spectral ellipsometer in the wavelength range of 0.5 to 1.5 μm. The refractive index in the mid-infrared band was obtained by fitting the measured data with the Sellmeier equation. The refractive index *n_s_* of amorphous silicon was expressed as

(2) $$n_{s}^{2}=1.802+\frac{6.496\lambda^{2}}{\lambda^{2}-0.3488^{2}}+\frac{0.01433\lambda^{2}}{\lambda^{2}-264.4^{2}}$$

where *λ* represents the wavelength of the incident light. When the wavelength was 4.6 μm, the refractive index of our amorphous silicon was 2.9, as shown in Figure 1c.

To obtain the desired phase gradient for polarization beam combining, the phase shifts of the unit cells were theoretically simulated. The reflective structure employed in this work is an Au-SiO_2_-Si configuration. The Au layer acts as a perfect reflector, while the top Si pillars serve as phase modulators. The SiO_2_ layer functions as a low-refractive-index dielectric spacer. These three layers collectively form a Fabry-Perot (F-P) resonant cavity. Through structural design, incident light can resonate within the cavity. This resonant enhances reflection efficiency and enables effective light manipulation using an ultrathin metasurface. The eigenmodes of the resonator are determined jointly by the thickness and refractive index of the SiO_2_ layer. Furthermore, the SiO_2_ layer of an appropriate thickness provides the necessary space for phase accumulation. Through simulation and analysis, we determined the optimal thickness of the SiO_2_ layer. In the simulations, the central wavelength of the incident light was 4.6 μm, the refractive indices of SiO_2_ and α-Si were 1.39 and 2.9, respectively, and the thicknesses of the three layers, H_1_, H_2_, and H_3_, were set to 2.3 μm, 0.9 μm, and 0.3 μm, respectively. The length (L) and width (W) of the rectangular pillars were varied from 0.4 μm to 1.4 μm. The periodic boundary conditions were applied along the *x*- and *y*-directions of the unit cell. A perfectly matched layer was set in the *z*-direction. And the incident wave was a plane wave in the *xoz* plane.

**Figure 2:**
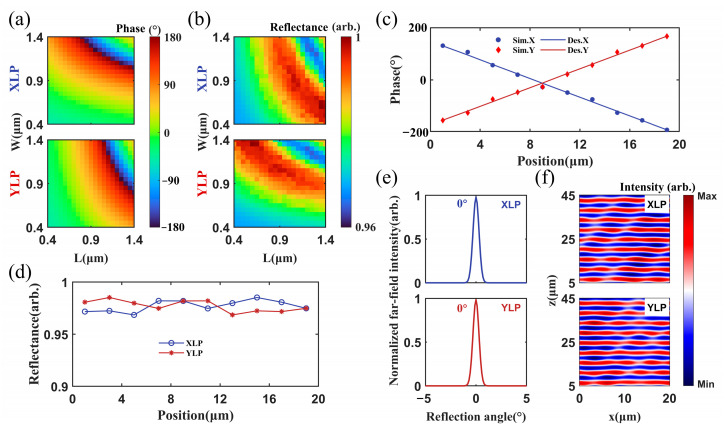
Design results of the metasurface polarization beam combiner. (**a**) Phase shifts and (**b**) reflectance of the unit cells when the L and W of the pillar were varied from 0.4 μm to 1.4 μm for the XLP and YLP incident lights, (**c**) desired and designed phase-shift distribution of a super cell for the XLP and YLP incident lights, (**d**) reflectance of the 10 selected discrete unit cells of the XLP and YLP incident lights, (**e**) normalized far-field intensity and (**f**) near-field wavefront distribution for the XLP and YLP lights incident at −13.3° and 13.3°.

The phase and amplitude responses of the unit cells are shown in Figure 2a,b, respectively. For both XLP and YLP lights, the phase shifts with different L and W covered −180° to 180°. And the reflectivity of all unit cells is higher than 96%.

Based on the phase shifts of the unit cells, 10 discrete unit cells were selected to satisfy the phase gradient requirements of Equation (2). For the XLP light, the metasurface should introduce a phase decrease along the positive *x*-direction with the gradient of −36°. Conversely, for the YLP light, the phase gradient along the positive *x*-direction should be 36°. Using the particle swarm optimization (PSO) algorithm, the dimensions of the pillars for the 10 discrete unit cells at different positions along the *x*-axis were selected. The results are listed in Table 1. The corresponding phase shifts are compared to the theoretical calculations in Figure 2c. The solid lines represent the theoretical calculations, and the scatter points indicate the phase shifts of the selected unit cell. They coincided with each other quite well. Figure 2d presents the reflectance of the selected 10 unit cells. It exceeds 0.97. The design results demonstrated that the phase introduced by the selected unit cells matched the target phase profiles for both XLP and YLP incident lights, and a high working efficiency of over 97% was expected.

Using the unit cells in Table 1, we simulated the reflectance of the collimated XLP and YLP incident lights with a wavelength of 4.6 μm. The two beams were incident on the metasurface at angles of −13.3° and 13.3°, respectively. Figure 2e,f show the normalized far-field intensities and the near-field distributions of the reflected waves. In Figure 2e, the reflection directions for both XLP and YLP incident beams occur at 0°, with the ultra-high reflectance of 97.8% and 98.4%, respectively. The simulation results confirmed that the metasurface polarization beam combiner had ultra-high combining efficiency. Notably, the high efficiency had nothing to do with the complicated surface coating. When the incident waves are plane waves, the reflected wavefronts for both XLP and YLP incident beams are shown in Figure 2f. The reflected field exhibited a superposition of plane waves, with the component in the beam combining direction being dominant. The normal direction of reflected field was perpendicular to the metasurface and aligned with the positive *z*-axis. This indicated that the reflection angle was 0°. To quantitatively evaluate wavefront distortion, we calculated the root mean square (RMS) value of the wavefront at *z* = 10 μm. The corresponding RMS values for XLP and YLP were 0.0052 and 0.0051, respectively. These simulation results demonstrated that the designed anomalous reflection metasurface was an effective polarization beam combiner.

## 3. Results and Discussion

**Figure 3:**
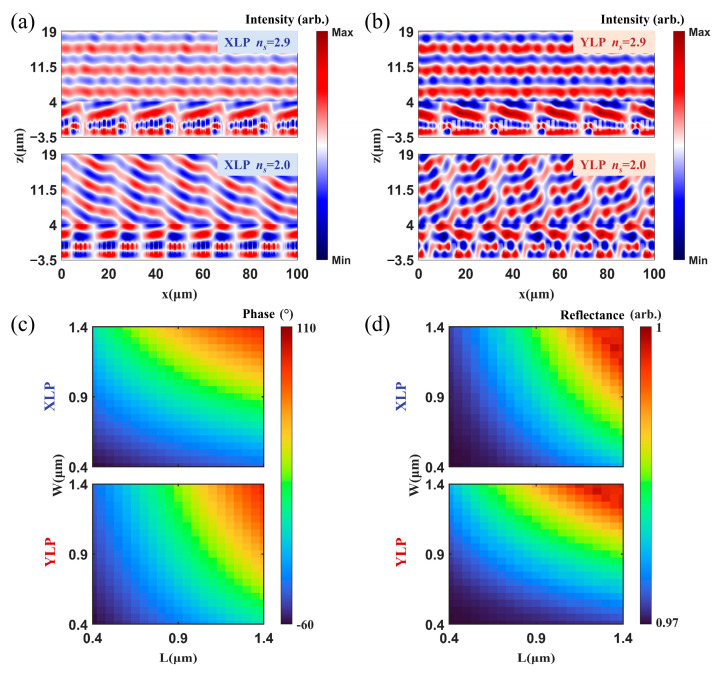
Simulation results of the metasurface performances when the refractive index of the α-Si pillars was 2.0 and 2.9. Near-field optical intensity distribution for the (**a**) XLP and (**b**) YLP lights incident with −13.3° and 13.3°, (**c**) Phase shifts and (**d**) reflectance of the unit cells when the L and W of the pillar were varied from 0.4 μm to 1.4 μm for the XLP and YLP incident light, when the refractive index of the α-Si pillars was 2.0.

The influences of the refractive index of α-Si on the reflection properties of the metasurface were investigated theoretically. Figure 3a,b show the optical field distributions of the XLP and YLP incident lights. For *z* < 0, the optical field distribution corresponds to the field within the metasurface structure. For *z* > 0, the distributions are the incident (*z* < 4 μm) and reflected fields (*z* < 4 μm). When the refractive index of α-Si was 2.9, the optical field was confined well inside the pillars, and the equiphase surfaces of both incident and reflected lights were planar, whose normal directions were consistent with the direction defined by the incident and reflected angles. In comparison, when the refractive index of α-Si was 2.0, the coupling between the neighbored pillars was evident, especially for the YLP incident light. In addition, most incident light was specularly reflected; only 4.4% was directed into the reflection direction. This was due to the inefficient phase shift coverage (−60° to 110°), as shown in Figure 3c. However, the total reflectivity of the unit cells still remained higher than 96.5%, benefiting from the high reflection induced by the Au bottom layer (Figure 3d).

The reflection properties of the metasurface polarization beam combiner across a 600 nm bandwidth were investigated by theoretical simulations.

**Figure 4:**
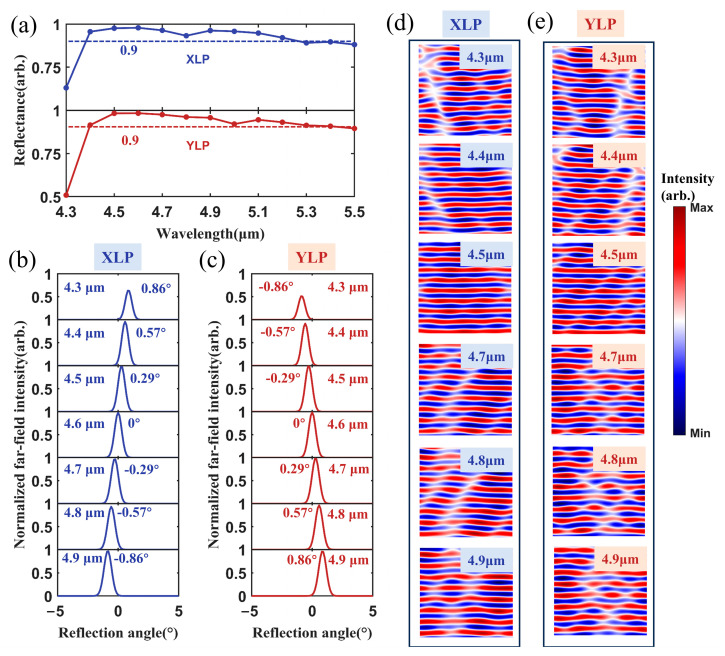
Reflectance of the metasurface polarization beam combiner across the 4.3–5.5 μm wavelength range. (**a**) Reflectance of the incident XLP and YLP lights within the wavelength range of 4.3–5.5 μm. Normalized far-field intensities of the (**b**) XLP and (**c**) YLP incident light across the 4.3–4.9 μm band, near-field wavefront distributions of the (**d**) XLP and (**e**) YLP incident lights in the 4.3–4.9 μm wavelength range.

Figure 4a shows the reflectance of the metasurface for the XLP and YLP incident lights, whose wavelength was varied from 4.3 μm to 5.5 μm. The reflectance remains above 90% within the 4.4~5.4 μm spectral range. The average reflectance of the XLP incident light was 94.4%, with a maximal reflectivity of 97.8% at 4.6 μm. For the YLP incident light, the average reflectance was 94.5%, with a maximum of 98.4% at 4.6 μm. Furthermore, we analyzed the normalized far-field intensity distributions and near-field distributions of the reflected beams across the 4.3~4.9 μm wavelength range. Figure 4b,c show the simulation results. The reflection angles decreased with the increasing incident laser wavelength for the XLP incident beam. The reflection angles increased with the increasing incident laser wavelength for the YLP incident beam. The reflection angle was 0° when the laser wavelength was 4.6 μm, no matter the incident laser polarization. The results indicate that the metasurface polarization beam combiner had a spectral dispersion of 0.05 mrad/nm. The unique dispersion characteristic revealed that the metasurface possessed the potential for spectro-polarimetric beam combining. The power of the combined beam could be significantly boosted by the single component. Figure 4d,e present the near-field distributions for the XLP and YLP incident lights. The reflected wavefronts for both polarizations remained planar. The weak wavefront distortion was observed only when the wavelength of the incident beam was 4.3 μm.

**Figure 5:**
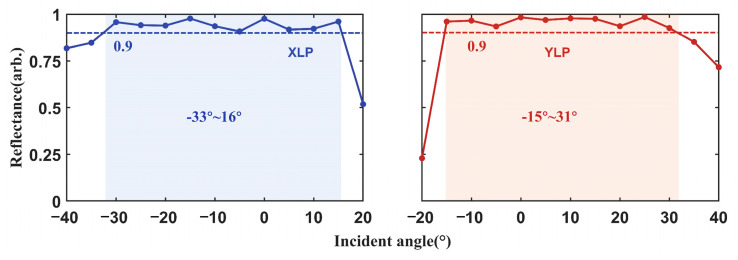
Reflectance of the incident XLP and YLP lights within the incident angles of −40°–20° and −20°–40°.

Additionally, we analyzed the reflectance for XLP and YLP light at different incident angles at 4.6 μm. The results are presented in Figure 5. The incident angle ranges were set from −40° to 20° for XLP and from −20° to 40° for YLP. For XLP, the reflectivity remains above 90% for incident angles between −33° and 16°. For YLP, reflectivity above 90% is maintained for incident angles between −15° and 31°.

These simulation results demonstrated that the metasurface polarization beam combiner maintained high beam combining efficiency across a wide spectral range from 4.4 μm to 4.9 μm. Within the whole spectral range, the wavefront distortion of the reflective beam was negligible. It ensured a nearly the same combined beam quality as that of the two incident beams.

**Figure 6:**
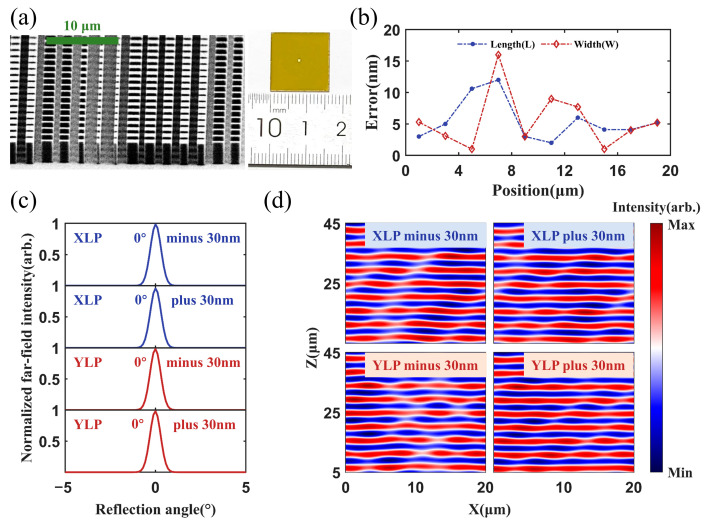
Metasurface combiner sample and error analysis. (**a**) SEM image of the fabricated sample. (**b**) The error results from fabrication deviations in unit cells. (**c**) Normalized far-field intensity and (**d**) near-field reflected wavefront for the XLP and YLP incident beams with ±30 nm fabrication deviations.

A metasurface beam combiner sample was fabricated using the optimized unit cell geometric parameters listed in Table 1. Figure 6a shows the photo and the scanning electron microscope (SEM) image of the fabricated sample. The fabrication errors in the geometric dimensions of the silicon nanopillars were statistically analyzed. The statistical results are presented in Figure 6b. The length and width errors were smaller than 20 nm. We simulated the metasurface performances with fabrication errors of ±30 nm. The wavelength of the incident light was set to 4.6 μm. Figure 6c and Figure 6d show the normalized far-field intensity and the wavefront profiles of the reflected lights with the XLP and YLP, respectively. The results indicated that even with the fabrication errors, the combiner could still efficiently reflect the incident beams to the 0° direction. A reflectivity greater than 98% was maintained under all fabrication error conditions, except for a slight reduction to 95% observed for the XLP light under positive tolerance. No obvious change in the reflected light wavefronts is observed in Figure 6d. The results demonstrated that the designed metasurface exhibited robust reflection performance against fabrication errors.

**Figure 7:**
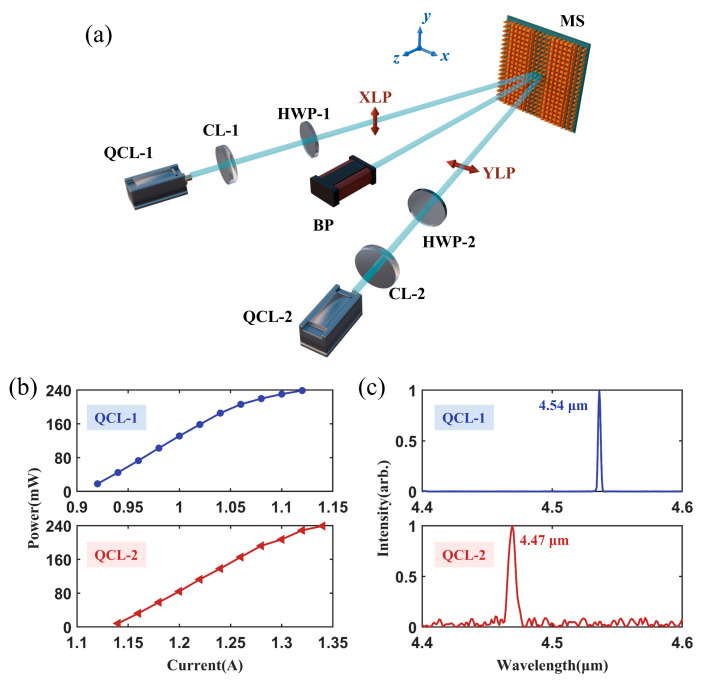
Properties of mid-infrared QCLs. (**a**) Schematic of the optical path for the metasurface PBC experiment. (**b**) Output power versus injection current for QCL-1 and QCL-2. (**c**) Emission spectra of QCL-1 and QCL-2 with the output power of 50 mW.

The experimental setup for the metasurface PBC is illustrated in Figure 7a. Two QCLs (QCL-1 and QCL-2) operated around 4.6 μm were used in our experiments. The output beams of the QCLs were collimated by aspheric lenses (CL-1 and CL-2). After that, the Half-wave plate (HWP-1 and HWP-2) was used to control the polarization. The two collimated beams were incident on the metasurface beam combiner (MS) at angles of −13.3° and 13.3°, respectively.

The measured properties of the two QCLs are summarized in Figure 7b,c. Figure 7b shows the output power versus injection current. The maximal output powers of both QCLs were 240 mW. Figure 7c shows the lasing spectra of the two QCLs. The central wavelengths of the QCL-1 and QCL-2 were 4.54 μm and 4.47 μm, respectively.

**Figure 8:**
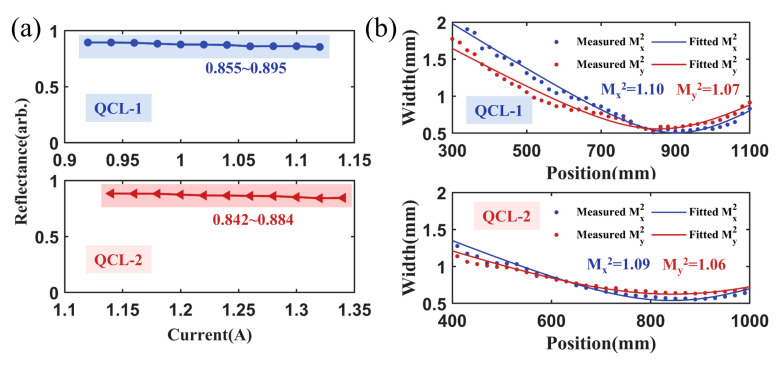
Reflection properties of the fabricated metasurface. (**a**) Reflectance of the metasurface with different injection currents for QCL-1 and QCL-2. (**b**) Beam quality factor *M*^2^ of the reflected QCL-1 and QCL-2.

When the output power of the QCLs was 50 mW, the reflected powers were 44.7 mW for QCL-1 and 44.2 mW for QCL-2. The corresponding reflectance was 89.5% and 88.3%, respectively. As the output power of the QCL increased, the reflectance for QCL-1 decreased from 89.5% to 85.5%; while that of QCL-2 decreased from 88.4% to 84.2%, as presented in Figure 8a. Compared to the simulation results, the relatively low efficiency might be caused by the imperfect linear polarization of the QCLs, the transmission losses of the optical components and the diffraction loss induced by the finite aperture of the metasurface (5 mm × 5 mm). Figure 8b presents the *M^2^* of the reflected beams. For QCL-1, the *M*^2^ in the fast and slow axes were 1.10 and 1.07, respectively. For QCL-2, the values were 1.09 and 1.06, respectively.

**Figure 9:**
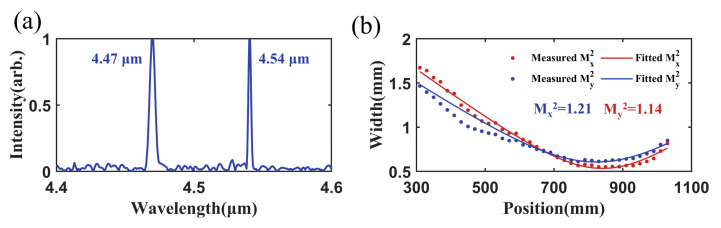
Experimental results of PBC. (**a**) Spectrum and (**b**) *M*^2^ of the combined beam.

The experimental results of the polarization beam combining are shown in Figure 9. The spectrum of the combined beam is presented in Figure 9a. The spectrum contained two distinct wavelength peaks located at 4.47 μm and 4.54 μm. Compared to the spectra of the QCLs, neither the central wavelength nor the spectral width shows significant changes, indicating that the metasurface combiner did not alter the spectral profile. When the output powers of both QCLs were 50 mW, the power of the combined beam was 88.9 mW, corresponding to a polarization combining efficiency of 88.9%. The *M*^2^ measurement results of the combined beam are shown in Figure 9b. The *M*^2^ values along the fast and slow axes were 1.21 and 1.14, respectively. Compared to those of the individual QCL, no noticeable degradation was observed.

These experimental results demonstrated that the metasurface polarization combiner had high combing efficiency, small wavefront distortion and broad operating spectral linewidth. The high efficiency did not depend on the complicated coatings. It represented a promising alternative to conventional bulk polarization beam combiners and offered enhanced design flexibility.

## 4. Conclusions

In this work, we proposed a polarization beam combiner based on a reflection metasurface operating in the mid-infrared band. The polarization beam combiner was composed of 2 μm × 20 μm periodically arranged supercells, which were fabricated on a silicon substrate. Each supercell contained 10 discrete 2 μm × 2 μm unit cells, which were composed of a Au bottom layer, a SiO_2_ spacer, and an amorphous silicon pillar. To minimize the loss and provide optical field confinement, α-Si pillars with a high refractive index of 2.9 were fabricated. The lengths and widths of the α-Si nanopillars were optimized for operation at 4.6 μm. When the XLP and YLP lights were incident at angles of −13.3° and 13.3°, respectively, the reflection angles of both beams were 0°. The simulated reflectance of the XLP and YLP lasers was 97.8% and 98.4%. In addition, the metasurface beam combiner exhibited a broad, high-efficiency working spectral band from 4.4 μm to 4.9 μm. Within the whole band, the average reflectance of the XLP and YLP lights was 96.2% and 96.3%, respectively. All the materials applied in the metasurface had a high laser damage threshold and good thermal conductivity. This made the metasurface beam combiner suitable for high-power applications. Uniquely, the metasurface beam combiner had spectral dispersion. The results revealed that the metasurface possessed the potential for spectro-polarimetric beam combining. When multiple incident lasers had different wavelengths and linear polarizations, they could be combined coaxially with high efficiency. The outputs of two QCLs with central wavelengths of 4.47 μm and 4.54 μm were successfully combined by our fabricated metasurface beam combiner. The overall beam combining efficiency of 88.9% was obtained with 100 mW input power. The beam quality factors *M^2^* of the combined beam along the fast and slow axes were 1.21 and 1.14, respectively. The experimental results demonstrated that the metasurface beam combiner had high efficiency, low inducing phase distortion and broad operation bandwidth.

In addition, metasurface-based beam combiners offer the advantages of high design flexibility and strong compatibility with standard CMOS fabrication processes. Compared to Brewster plates, the polarization device proposed in this work can be fabricated using micro–nano-manufacturing techniques without requiring additional coating processes. Our device achieves beam combining across a bandwidth of 4.4–5.4 μm, sufficient to cover the spectral width of typical QCLs. In contrast to birefringent crystals, the incident angles of the two combined beams in our design can be flexibly adjusted by engineering the metasurface structure, offering greater simplicity and adaptability in optical path design.

## Figures and Tables

**Table 1:** The length and width of the selected α-Si pillars at different *x* positions. Note: The N1 corresponds to the position of the first unit cell in the supercell, N2 to the second, and so on.

Position of Si	N1	N2	N3	N4	N5	N6	N7	N8	N9	N10
Length *L* (μm)	0.4	0.4	1.425	1.275	1.125	1.025	0.925	1.4	1.2	0.725
Width *W* (μm)	1.2	1.4	0.925	1.025	1.125	1.275	1.425	0.4	0.4	0.725

## Data Availability

The data presented in this study are available from the corresponding author upon reasonable request.
